# The effect of hydrogen-rich water consumption on premenstrual symptoms and quality of life: a randomized controlled trial

**DOI:** 10.1186/s12905-024-03029-8

**Published:** 2024-03-26

**Authors:** Menekşe Nazlı Aker, İlknur M. Gönenç, Dilan Çalişici, Menekşe Bulut, Duried Alwazeer, Tyler W. LeBaron

**Affiliations:** 1 Nursing Faculty, Ankara University, Ankara, Turkey; 2 Department of Food Engineering, Iğdır University, 76000 Iğdır, Turkey; 3 Department of Nutrition and Dietetics, Faculty of Health Sciences, Iğdır University, 76000 Iğdır, Turkey; 4 Research Center for Redox Applications in Foods (RCRAF), Igdir University, 76000 Igdir, Turkey; 5 Application, and Research Center, Innovative Food Technologies Development, Igdir University, 76000 Igdir, Turkey; 6 Department of Kinesiology and Outdoor Recreation, Southern Utah University, Cedar City, UT 84720 USA; 7 Molecular Hydrogen Institute, Enoch, UT 84721 USA

**Keywords:** Premenstrual syndrome, Quality of life, Molecular hydrogen, Hydrogen-rich water, Questionnaires

## Abstract

**Background:**

Premenstrual syndrome (PMS) consists of psychiatric or somatic symptoms negatively affecting the daily life. PMS treatment can involve the use of complementary-alternative approaches. Hydrogen-rich water (HRW) has antioxidant and anti-inflammatory properties that may treat PMS. This study aimed to investigate the effect of drinking HRW on the severity of premenstrual symptoms and the quality of life of women who suffer from PMS.

**Methods:**

This study is a randomized controlled trial. Participants were randomized into two groups (intervention group=33, control group=32) using the block randomization method. Participants were requested to consume 1500-2000 mL of HRW daily in the intervention group and drink water in the placebo group. Participants began drinking either HRW or placebo water from day 16 of their menstrual cycle until day 2 of the following cycle for three menstrual cycles. The research data were collected using a Demographic Information Form, Premenstrual Syndrome Scale (PMSS), and Short form of the World Health Organization Quality of Life Questionnaire (WHOQOL- BREF).

**Results:**

The intervention group had significantly lower mean scores than the control group in both the first and second follow-ups on the PMSS (*P*<0.05). In the first follow-up, the intervention group had significantly higher mean scores in the Physical Health and Psychological domains of the WHOQOL-BREF compared to the control group (*P*<0.05). Group × time interaction was significant for PMSS (*F* = 10.54, *P*<0.001). Group × time interaction was insignificant for WHOQOL- BREF (*P*>0.05).

**Conclusions:**

The consumption of HRW reduces the severity of premenstrual symptoms and improves individuals' quality of life in physical and psychological domains.

## Introduction

Many women of reproductive age experience somatic and mental problems during the menstrual period [1]. Premenstrual syndrome (PMS) consists of psychiatric or somatic symptoms that develop during the luteal phase of the menstrual cycle, negatively affecting the individual's daily life [2]. In a systematic review examining the frequency of PMS in Turkey, the prevalence has been reported as 52.2% [3]. PMS, being such a widespread health issue, can negatively impact women's quality of life [1, 4–7], thus highlighting the importance of PMS treatment.

The pathogenesis of PMS is complex and not fully understood [8]. It is believed that the menstrual cycle and the underlying hormones influence the psychophysiological and biological processes related to PMS [9]. However, some studies have not shown differences in hormone levels between healthy and PMS women [9, 10]. One hypothesis suggested that the response alternated based on exposure or withdrawal from allopregnanolone, a progesterone metabolite and an agonist of gamma-aminobutyric acid [11]. Scientists are currently exploring another possible cause of PMS, which is an abnormal inflammatory response to different types of stimuli, such as biological or physical factors. This response may lead to oxidative stress, the imbalance between reactive oxygen species (ROS) production and their inactivation by antioxidants. Both inflammation and oxidative stress are being studied as possible factors in the development of PMS [8]. Therefore, it is believed that reducing inflammation and oxidative stress would be effective in the treatment of PMS. PMS treatment focuses on alleviating physical and psychiatric symptoms and can involve various dietary supplements and complementary alternative approaches [2]. Thus, one can assume the potential of hydrogen-rich water (HRW) to be benefit in the treatment of PMS.

Molecular hydrogen, the smallest molecule in the universe, is rapidly absorbed into the circulatory system following ingestion of HRW [12]. Hydrogen can penetrate biomembranes and permeate into the cytosol, mitochondria, nucleus, and blood-brain barrier. Consequently, tissues can rapidly absorb hydrogen [13, 14]. Molecular hydrogen (H_2_) is a light gas yet a selective antioxidant and has recently been recognized as a novel therapeutic agent [13]. H_2_ has been proposed as a potential treatment for certain neuromuscular disorders, cardiometabolic diseases, and types of cancer, primarily functioning as an anti-inflammatory agent and a selective antioxidant [15].

Molecular hydrogen can upregulate the enzymatic antioxidant systems, including glutathione peroxidase, superoxide dismutase, and catalase [14]. H_2_ can selectively scavenge the most harmful reactive oxygen/nitrogen species, e.g., hydroxyl radical and peroxynitrite, without affecting beneficial ones, e.g., hydrogen peroxide or nitric oxide [13, 15].

The consumption of HRW has been reported to be beneficial for daily life [16]. Due to the antioxidant and anti-inflammatory properties of HRW, the present study investigated if HRW can be used to treat PMS, a condition involving inflammation and oxidative stress in its pathophysiology. No study has been found in the literature investigating the effects of HRW consumption on PMS symptoms and quality of life. To the best of our knowledge, this study represents the first investigation conducted in this area.

### Aim

This study aimed to determine the effect of HRW consumption on premenstrual symptoms and the quality of life of women (students) experiencing premenstrual syndrome.

### Hypotheses

H_0_1 There is no significant difference in the Premenstrual Syndrome Scale scores between participants consuming HRW and those in the control group.

H_0_2 There is no significant difference in the Short form of the World Health Organization Quality of Life Questionnaire scores between participants consuming HRW and those in the control group.

## Materials and methods

### Design

**Fig. 1:**
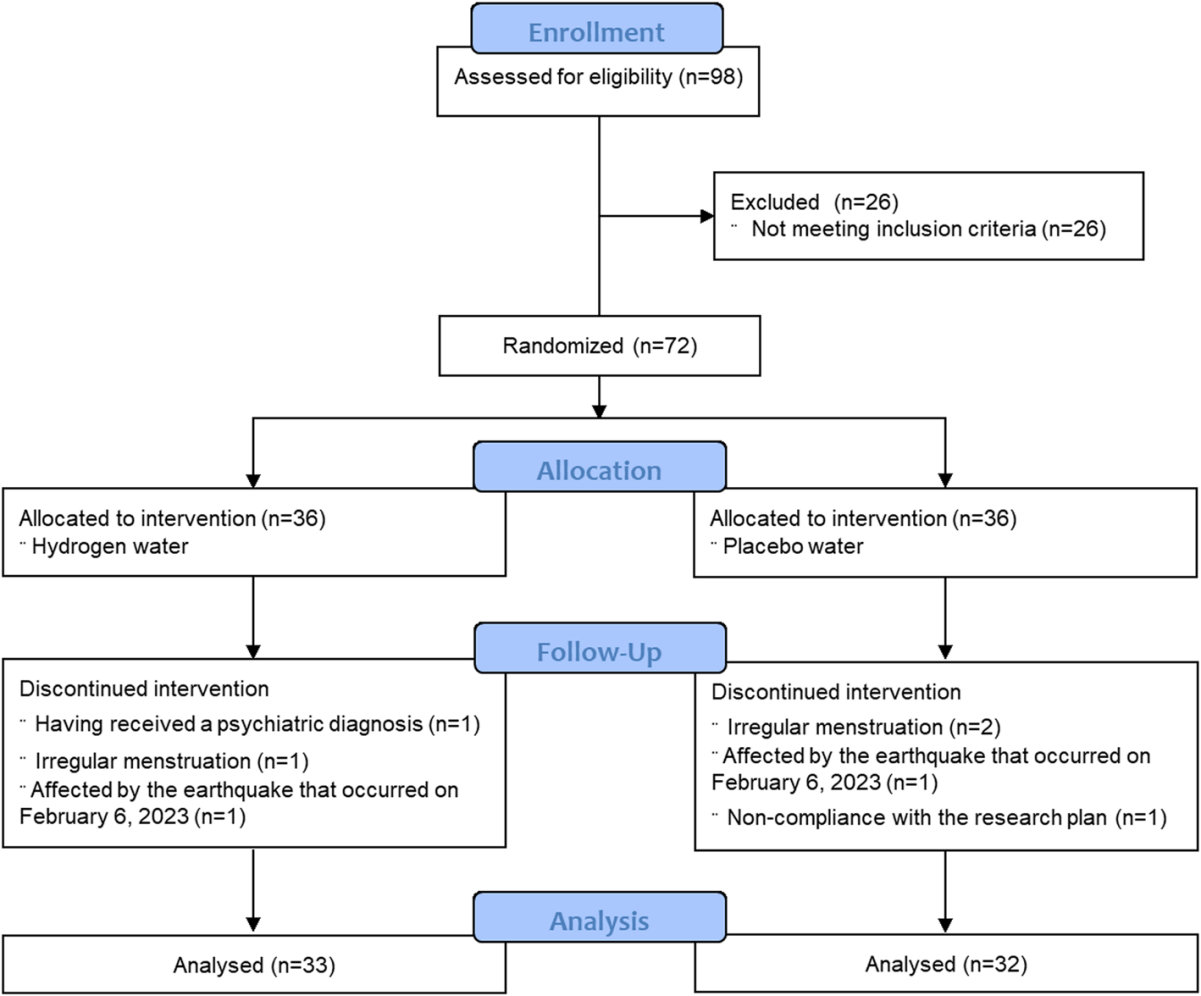
The study flowchart, according to CONSORT 2010

This study is a single-center, parallel-group, stratified, blocked, randomized, controlled experimental study. Every step of the study was conducted according to the Consolidated Standards of Reporting Trials (CONSORT) guidelines (Fig. 1). This study was registered in the Clinical Trials registry system (https://clinicaltrials.gov/study/NCT05556252#study-record-dates) with the registration number NCT05556252 on 27/09/2022.

### Population and sample

The study was conducted at the Faculty of Nursing, Ankara University (Turkey). The Faculty consists of departments of midwifery and nursing, with the majority of students being female. Initially, 775 volunteers were evaluated to assess those meeting the inclusion criteria for premenstrual syndrome (PMS) using the Premenstrual Syndrome Scale. Participants were classified as experiencing PMS if they scored 132 or above on the scale defined by Gençdoğan B. [17]. At this stage, it was found that 366 (47.2%) of the participants experienced PMS. Among these identified participants experiencing PMS, those willing to participate in the randomized controlled trial were evaluated based on the inclusion criteria for enrollment. The inclusion criteria for the study were having a menstrual cycle length within the normal range (21-35 days), regular menstruation in the last three cycles, scoring 132 or above on the Premenstrual Syndrome Scale [17], not receiving medical treatment for PMS, and not having any psychiatric diagnosis. The exclusion criteria included having received a psychiatric diagnosis, having any gynecological disease (such as abnormal uterine bleeding, myoma, ovarian cyst, etc.), using a hormonal contraceptive medication, non-compliance with the research plan by the participants, having pregnancy and participants initiating PMS treatment during the study period.

The study consisted of intervention and control groups. Individuals consuming HRW were assigned to the intervention group, while individuals consuming placebo water were assigned to the control group. G*Power 3.1.9 software was used to calculate the sample size for this study. No studies were found in the literature investigating the effects of HRW on premenstrual syndrome. Therefore, the study conducted by Taavoni et al. (2014) [18], which evaluated the effects of Royal Jelly on premenstrual syndrome, was used as a basis, and the minimum required sample size for a power of 0.9 and an alpha level of 0.05, with an effect size (d) of 0.73, was determined to be 33 for both the intervention and control groups, totaling 66 participants. A total of 98 individuals agreed to participate in the study. Among them, 10 had menstrual irregularities, 6 had a psychiatric diagnosis, 7 had a gynecological disease diagnosis, and 3 were using oral contraceptives. Therefore, a total of 72 volunteers were included in the study.

The volunteers were sorted in ascending order according to their student numbers. The block randomization method was utilized for assigning volunteers to groups in accordance with this sequence. The randomized block procedure was performed as follows: (a) block size of 6 was selected; (b) subjects were calculated as having 12 conditions (ABBBAA, ABABBA, BABBAA, BBABAA, BAABBA, ABBAAB, BBBAAA, ABABBA, BABAAB, BABABA, AABBAB and BBABAA); and (c) blocks were randomly selected to determine the assignment of all 72 participants, with an allocation ratio of 1:1. The study was completed with 33 participants in the intervention group and 32 participants in the control group (Fig. 1).

There was no statistical difference in the descriptive characteristics of the intervention and control groups (Table 1).

**Table 1:** Distribution of participants' descriptive characteristics (*n=*65)

**Variable**	**Intervention Group (*n=*33)**	**Control Group (*n=*32)**		
	**Mean±SD**	**Mean±SD**	**Total Mean±SD**	**Test value/*P*-value**
Age (years)	20.79±1.34	20.41±.84	20.60±1.129	t=1.372 *P*=0.175
Age at menarche (years)	12.76±1.20	12.94±.91	12.85±1.06	t=-0.679 *P*=0.500
Menstrual cycle duration (days)	27.33±2.88	26.44±3.29	26.89±3.10	t=1.169 *P*=0.247
Menstruation duration (days)	5.55±1.09	5.91±1.06	5.72±1.08	t=-1.352 *P*=0.181
Body Mass Index	22.02±4.32	23.03±4.26	22.52±4.29	t=-0.949 *P*=0.346
	**n (%)**	**n (%)**	**Total n (%)**	
Place of residence				
Student dormitory	16 (48.5)	16 (50.0)	32 (49.2)	X=3.041 *P*=0.219
With family	11 (33.3)	12 (37.5)	23 (35.4)	
In a student's house	6 (18.2)	4 (12.5)	10 (15.4)	

*Abbreviations*: *t* Independent t Test, *X* Chi Square

The study was conducted as a double-blind trial. For participant blinding, participants in the control group were provided with a bottle resembling a hydrogen water generator. HRW is colorless, odorless, and tasteless. Therefore, participants were not expected to discern the properties of the water they consumed [19]. Additionally, to prevent bias during the data collection process, the data collection forms used in the study were administered by a researcher unaware of the participants' group allocations.

### The implementation of the study

The following interventions were implemented in the study groups.

#### Intervention group

Participants in this group were provided with HRW for consumption. Each participant was given a 500 mL portable hydrogen water generator (Novasmart, Turkey). Additionally, they were provided with drinking water (Sırma, Turkey) (0.5 L per PET bottle with 2.0 L per day) to prepare HRW. After filling the portable hydrogen water generator with drinking water, it was run for 5 minutes to achieve the saturation level of hydrogen [19, 20]. In a preliminary application conducted by researchers, it was observed that the H_2_ concentration of hydrogen-rich water (HRW) gradually decreased after preparation. Consistent with this observation, participants were instructed to consume HRW immediately after preparation [19]. Before starting the intervention, participants received training on how to use the hydrogen water generator. The consumption of HRW was individually tailored to each participant's menstrual cycle. Each participant commenced HRW consumption approximately on the 16^th^ day of their menstrual cycle, coinciding with the onset of the secretory phase, and continued until the 2^nd^ day of the menstrual phase. This pattern was maintained in the subsequent cycles. As part of the study, participants were instructed to consume 300-400 mL of HRW in the following time intervals: one hour before breakfast, one hour before lunch, two hours after lunch, one hour before dinner, and half an hour before bedtime. Thus, participants were requested to consume a minimum of 1500 mL and a maximum of 2000 mL HRW daily [21]. Since the application for changes in PMS symptoms should last for three menstrual cycles [1, 22], participants in this study were also provided with HRW for three cycles. A water intake tracking chart monitored participants' adherence to the program.

#### Control group

Participants in this group were provided with a flask similar to the hydrogen water generator but did not produce hydrogen or change the water properties. Participants in the control group (placebo) were instructed to consume the same amount of water (Sırma, Turkey) (0.5 L per PET bottle with 2.0 L per day) on the same days as the intervention group. A water intake tracking chart monitored participants' adherence to the program.

### Data collection

The research data were collected using a Demographic Information Form, Premenstrual Syndrome Scale, and Short form of the World Health Organization Quality of Life Questionnaire.

### Demographic information form

The form included questions to determine participants' socio-demographic characteristics and menstrual history. All participants completed this form after being assigned to their respective groups.

### The Premenstrual Syndrome Scale (PMSS)

The Premenstrual Syndrome Scale (PMSS), developed, validity and reliability study was conducted by Gençdoğan (2006) based on DSM-III and DSM-IV-R, is a five-point Likert scale consisting of 44 items that measure the severity of premenstrual symptoms. The scale includes nine subscales: depressive mood, anxiety, fatigue, irritability, depressive thoughts, pain, appetite changes, sleep changes, and swelling. The Total Score of the PMSS can range from a minimum of 44 to a maximum of 220. An increase in the score indicates an increase in the intensity of PMS symptoms. Participants scoring 132 and above on the scale are considered to have PMS. The total Cronbach's alpha reliability coefficient of the scale is 0.75 [17]. In this study, Cronbach's alpha reliability coefficient for the initial measurement is 0.87.

The measurement times for assessing the severity of PMS were determined based on the study conducted by [22]. All participants' premenstrual symptoms were evaluated three times: before starting the intervention (on days 7-10 of their last menstrual cycle), after the application of three cycles of HRW/placebo water (on days 7-10 of menstruation), and one month after the completion of HRW/placebo water application (on days 7-10 of subsequent menstrual cycles).

### Short form of the World Health Organization Quality of Life Questionnaire (WHOQOL- BREF)

In 1980, the World Health Organization (WHO) developed the WHOQOL-100 scale to define the quality of life and enable cross-cultural comparisons. The WHOQOL-BREF is a short form of 27 items derived from the WHOQOL-100 [23]. This form's Turkish validity and reliability study was conducted by [24]. The scale included 27 questions, each one scored on a scale of 1 to 5, with scores ranging from 0 to 100. The remaining questions were used to calculate scores for the physical health, psychological, social, and environmental domains, except for the first two general questions. Higher scores on the scale indicate a higher quality of life. The Cronbach's alpha values for the WHOQOL-BREF are as follows: physical health 0.83, psychological 0.66, social 0.53, and environment 0.73 [24]. In this study, Cronbach's alpha reliability coefficients for the initial measurement were calculated as follows: physical health 0.61, psychological 0.70, social 0.56, and environment 0.73.

The measurement times for WHOQOL-BREF were determined based on when the severity of PMS was assessed. All participants' premenstrual symptoms were evaluated three times: before starting the intervention (on days 7-10 of their last menstrual cycle), after the application of three cycles of HRW/placebo water (on days 7-10 of menstruation), and one month after the completion of HRW/placebo water application (on days 7-10 of subsequent menstrual cycles).

### Data analysis

The statistical analysis was performed using the SPSS Windows 24.0 software package, and a significance level of *P*<0.05 was considered. The normality of the data was assessed by examining the skewness and kurtosis values, and values within the range of ±2 were considered indicative of a normal distribution [25]. The Chi-Square statistic was utilized to test relationships between categorical variables. Independent Samples t-test was employed to compare the independent groups in terms of menstrual characteristics, PMSS, Physical health, Social domain, Environment domain scores, and Psychological domain scores at the initial two measurements. Repeated-measures MANOVA was used to examine significant differences in the PMSS, Physical health, Social domain, and Environment domain scores across time among the groups. For the comparison of the independent groups in the Psychological domain at the third follow-up measurement, the Mann-Whitney U test was conducted, while the Friedman test was utilized to assess time-dependent changes.

## Results

**Fig. 2:**
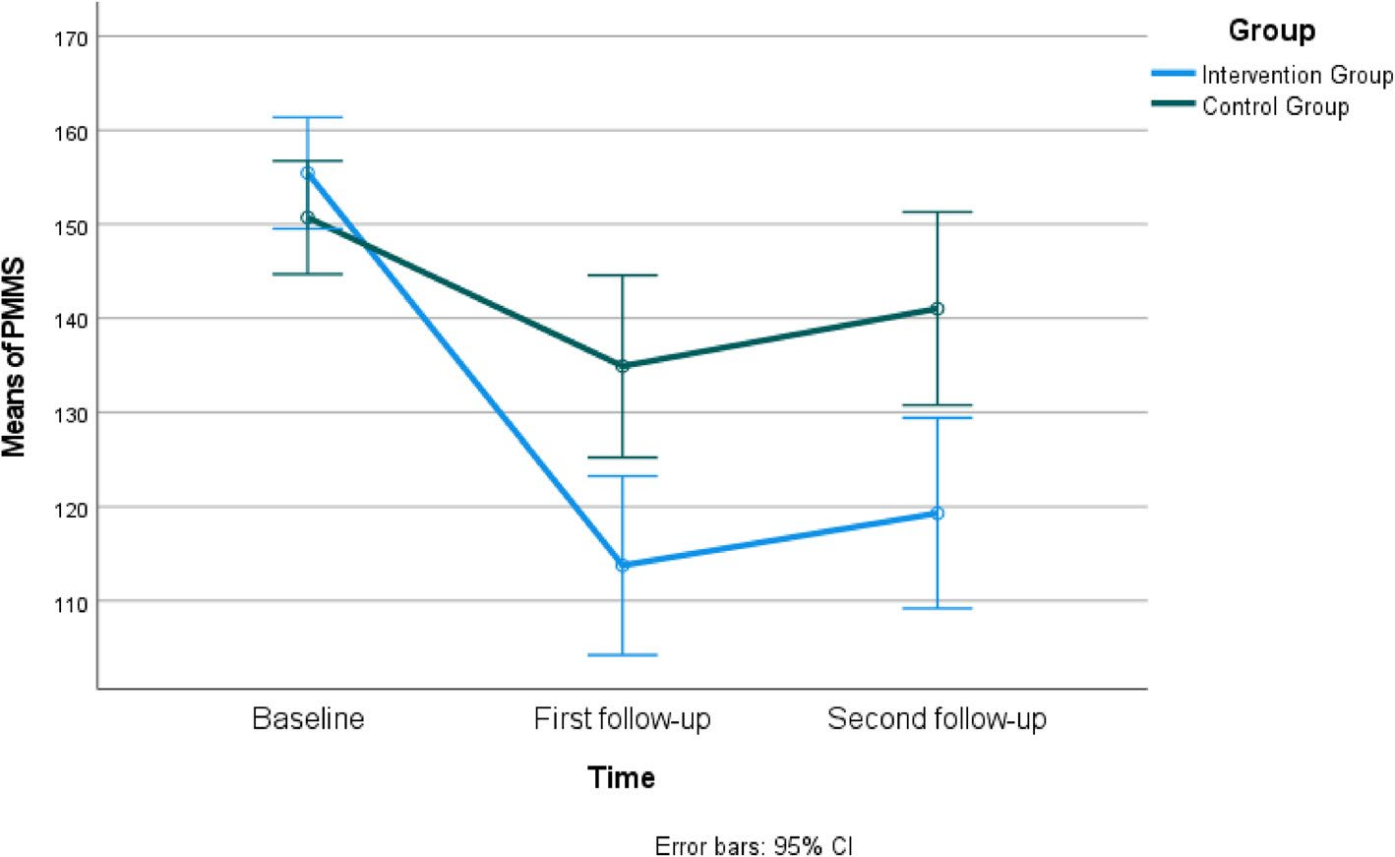
Estimated marginal means of PMSS

When comparing the mean PMSS scores between the intervention and control groups, it was found that the mean scores were not statistically different at the baseline (*P*>0.05), but in the first and second follow-ups, the intervention group had significantly lower mean scores compared to the control group (*P*<0.05) (Fig. 2). Group × time interaction was significant for PMSS (*F* = 10.54, *P*< 0.001) (Table 2).

**Table 2:** The comparison of participants' Premenstrual Syndrome Scale (PMSS) scores (*n=*65)

	**Intervention Group**	**Control Group**			
	**Mean±SD**	**Mean±SD**	**Test value/*P*-value**	**Cohen's d**	**95% Cl**
Baseline (a)	155.45±19.74	150.72±13.73	t=1.120 *P*=0.267	0.278	-3.716 to 13.188
First follow-up (b)	113.76±29.45	134.91±24.96	t=-3.118 *P*=0.003*	0.774	-34.702 to -7.596
Second follow-up (c)	119.30±33.36	141.03±23.84	t=-3.013 *P*=0.004*	0.748	-36.139 to -7.318
**Time**	*F* = 42.64 *P*< 0.001				
**Group**	*F* = 6.69 *P* = 0.012				
**Group × time**	*F* = 10.54 *P*< 0.001				

Mauchly's test of sphericity: Mauchly's W; 0.890, Approx. Chi-Square; 7.198, p;0.027. The Greenhouse-Geisser test was utilized due to the assumption that sphericity was unmet

*Abbreviations*: *t* Independent t Test, *F* Repeated-measures MANOVA

*P<0.05

**Fig. 3:**
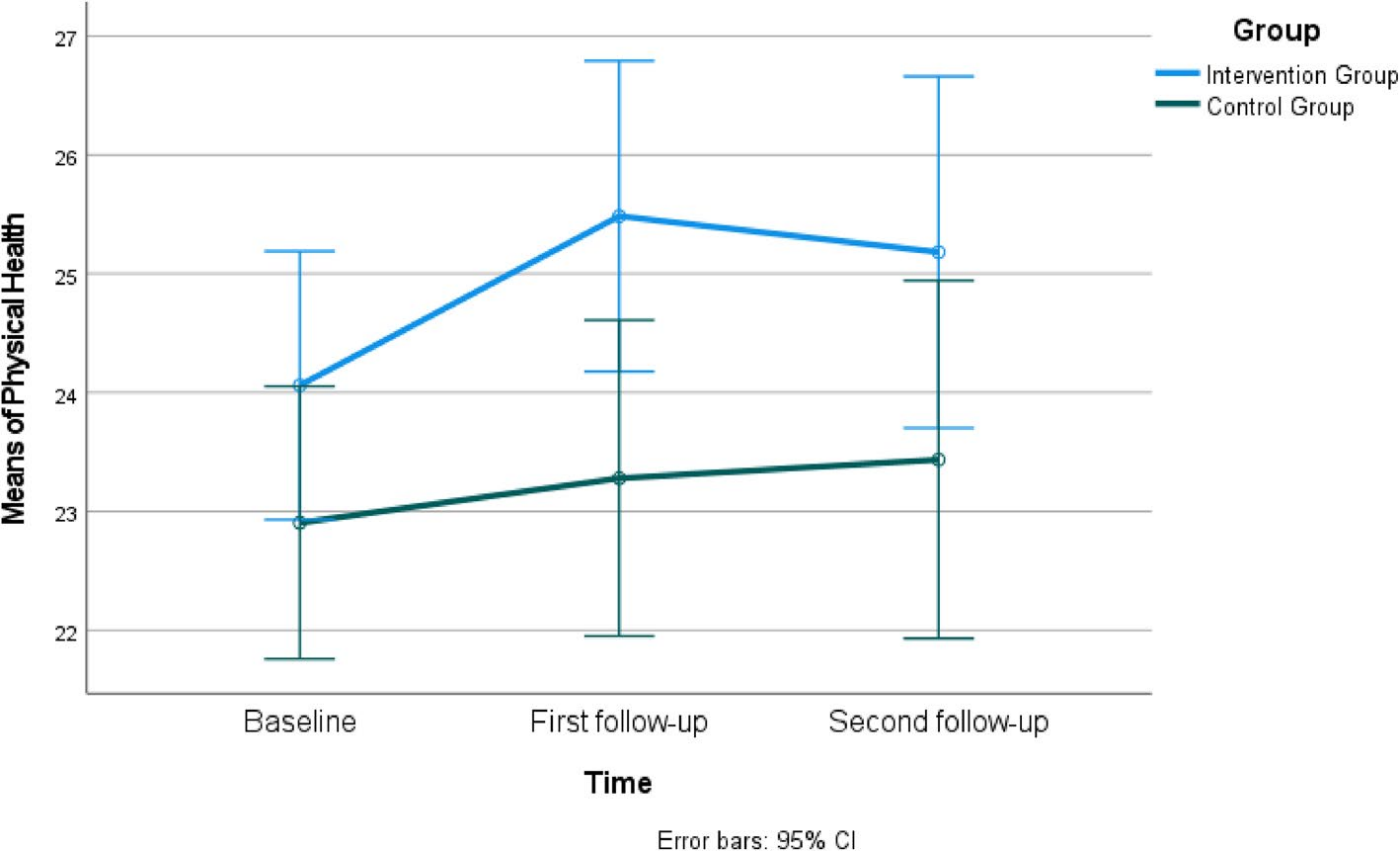
Estimated marginal means of physical health

**Fig. 4:**
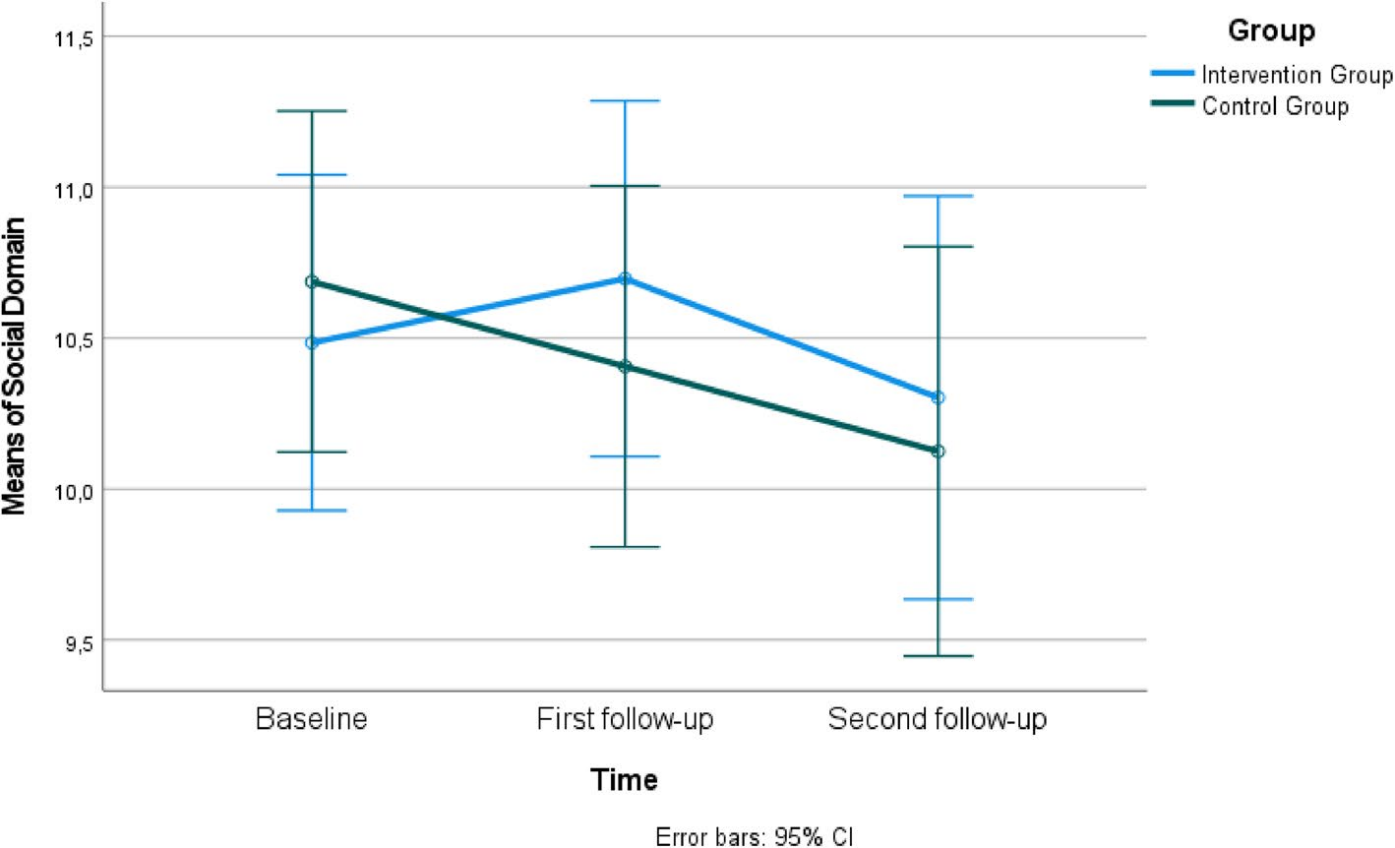
Estimated marginal means of social domain

**Fig. 5:**
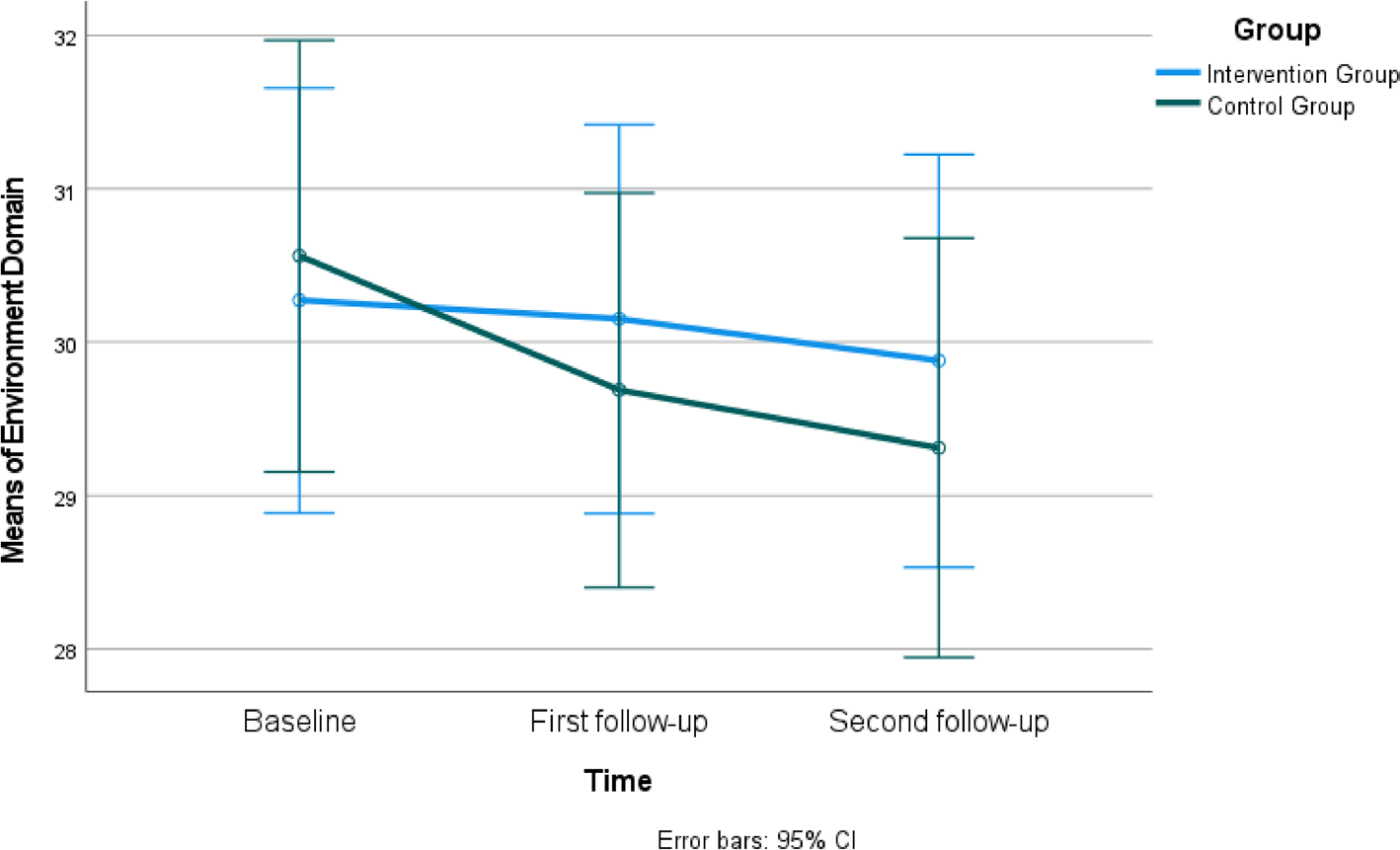
Estimated marginal means of environment domain

When comparing the mean scores of the WHOQOL- BREF between the intervention and control groups, it was found that the mean scores in all subscales were not statistically different at the baseline (*P*>0.05). However, in the first follow-up, the intervention group had significantly higher mean scores in Physical Health (Fig. 3) and median scores in the Psychological Domain compared to the control group (*P*<0.05) (Table 3). In the first follow-up, the scores in the Social Domain (Fig. 4) and Environment Domain (Fig. 5) were similar in both groups (*P*>0.05), while in the second follow-up, all domains had similar scores in both groups (*P*>0.05). Group × time interaction was not significant for WHOQOL- BREF (*P*>0.05) (Table 3).

**Table 3:** The comparison of participants' Short form of the World Health Organization Quality of Life Questionnaire (WHOQOL- BREF) scores (*n=*65)

**Physical Health**	**Intervention Group**	**Control Group**			
	**Mean±SD**	**Mean±SD**	**Test value/*P*-value**	**Cohen's d**	**95% Cl**
Baseline (a)	24.06±2.98	22.91±3.50	t=1.435 *P*=0.156	0.356	-0.454 to 2.762
First follow-up (b)	25.48±3.15	23.28±4.30	t=2.362 *P*=0.021*	0.586	0.339 to 4.068
Second follow-up (c)	25.18±4.74	23.44±3.70	t=1.651 *P*=0.104	0.410	-0.367 to 3.856
**Time**	*F*=2.02 *P*=0.143				
**Group**	*F*=5.28 *P*=0.025				
**Group × time**	*F*=0.56 *P*=0.554				
Mauchly's test of sphericity: Mauchly's W; 0.879, Approx. Chi-Square; 8.009, p;0.018. The Greenhouse-Geisser test was utilized due to the assumption that sphericity was unmet.					
**Psychological Domain**					
	**Mean±SD (Median, Min-Max)**	**Mean±SD (Median, Min-Max)**	**Test value/*P*- value**		
Baseline (a)	19.12±3.16 (19.5, 13-25)	18.13±3.18 (18.0, 11-24)	t=1.267 *P*=0.210	0.314	-0.575 to 2.568
First follow-up (b)	19.73±2.27 (18.5, 13-24)	17.88±2.64 (18.5, 13-22)	t=3.040 *P*=0.003*	0.754	0.635 to 3.070
Second follow-up (c)	18.67±3.03 (19.0, 7-23)	18.16±3.18 (19.0, 9-24)	U=472.5 *P*=0.463	0.164	-1.029 to 2.050
Test/* P*	X=1.702 *P*=0.427	X=0.698 *P*=0.705			
**Social Domain**					
	**Mean±SD**	**Mean±SD**	**Test value/*P*-value**		
Baseline (a)	10.48±1.70	10.69±1.49	t=-0.511 *P*=0.611	0.127	-0.996 to 0.590
First follow-up (b)	10.70±1.78	10.41±1.60	t=0.692 *P*=0.492	0.172	-0.549 to 1.130
Second follow-up (c)	10.30±2.08	10.13±1.74	t=0.374 *P*=0.710	0.093	-0.774 to 1.130
**Time**	*F* = 2.00 *P*=0.140				
**Group**	*F* = 0.60 *P*=0.807				
**Group × time**	*F* = 0.79 *P*=0.456				
Mauchly's test of sphericity: Mauchly's W; 0.987, Approx. Chi-Square; 0.842, p;0.656. The Sphericity Assumed test was utilized as the assumption of sphericity was met.					
**Environment Domain**					
	**Mean±SD**	**Mean±SD**	**Test value /*P*-value**		
Baseline (a)	30.27±4.88	30.56±2.76	t=-0.293 *P*=0.770	0.073	-2.257 to 1.677
First follow-up (b)	30.15±3.51	29.69±3.77	t=0.514 *P*=0.609	0.127	-1.341 to 2.269
Second follow-up (c)	29.88±4.22	29.31±3.47	t=0.590 *P*=0.557	0.146	-1.351 to 2.484
**Time**	*F* = 1.05 *P*=0.354				
**Group**	*F* = 0.13 *P*= 0.719				
**Group × time**	*F* = 0.33 *P*=0.717				
Mauchly's test of sphericity: Mauchly's W; 0.900, Approx. Chi-Square; 6.516, p;0.038. The Greenhouse-Geisser test was utilized due to the assumption that sphericity was unmet.					

*Abbreviations*: *t* Independent t Test, *F* Repeated-measures MANOVA, *X* Friedman test, *U* Mann-Whitney U test

*P<0.05

## Discussion

This study represents the first investigation into the effects of consuming HRW on premenstrual symptoms and quality of life in students experiencing premenstrual syndrome (PMS). Our findings indicate that the consumption of HRW in this study reduced PMS symptoms and improved quality of life, particularly in the physical health and psychological domains. No previous studies have been found in the literature examining the effects of HRW consumption on PMS. However, Zhang et al. (2021) have determined that supplements containing exogenous H_2_ play essential roles in regulating the homeostasis of sexual organs in females. H_2_ supplementation has been shown to alleviate ovarian injuries induced by ischemia/reperfusion (I/R) and certain drugs [26]. Additionally, it can mitigate premature ovarian failure (POF) and promote follicle development. Furthermore, H_2_ has decreased inflammation in the uterus [26]. It has been revealed that HRW decreased the malondialdehyde, cortisol, and testosterone in female rats with polycystic ovary syndrome [27]. Another report revealed that HRW alleviated epithelial degeneration and necrosis in muscle fibers of tissues in animal models [28].

Increased oxidative stress [29, 30] and decreased antioxidant capacity may occur in PMS. Imbalance in the oxidant/antioxidant systems has been reported as a potential cause or consequence of various stress symptoms in PMS [30]. As a therapeutic medical gas, molecular hydrogen possesses antioxidant properties and selectively scavenges cytotoxic reactive oxygen species (ROS) in tissues, thereby reducing tissue inflammatory events [31]. Based on this information, it is hypothesized that HRW consumption may reduce PMS by restoring the balance of oxidant/antioxidant systems and reducing excessive inflammation levels.

Another action mechanism reported that H_2_ acts similarly to estrogen derivatives by upregulating the endogenous expression of antioxidant enzymes and peptides as an antioxidant [32]. During the late luteal phase of the menstrual cycle, estrogen hormone levels are low in preparation for menstruation. These low levels appear to be a factor in the onset of PMS symptoms [33]. Consumption of HRW may positively benefit PMS by temporarily increasing estrogen concentration without significantly altering the menstrual cycle.

The presence of PMS in women negatively affects physical health, psychological health, and social relationships, leading to a decrease in overall quality of life [34]. Premenstrual symptoms can cause impairments in physical functioning and psychological health. Studies comparing women with and without PMS have shown that women with PMS experience adverse effects on the physical and psychological domains of quality of life [4, 35, 36]. No studies that specifically investigate the impact of HRW consumption on the quality of life in women with PMS have been encountered. However, Kang et al. (2011) demonstrated in their study that consumption of HRW for six weeks reduced reactive oxygen metabolites in the blood, maintained blood oxidation potential, and significantly improved quality of life scores during radiotherapy in cancer patients compared to those who received a placebo [37]. Mizuno et al. (2017) also reported that four weeks of HRW application in adult volunteers improved mood, reduced anxiety, enhanced autonomic nervous system function, and strengthened quality of life [38]. In the literature, the consumption of HRW has been reported to reduce fatigue in healthy individuals [38] and improve cognitive function and anxiety levels [39]. These findings indicate that HRW may be effective in enhancing quality of life.

## Conclusions

In conclusion, this study has demonstrated that consumption of HRW may reduce the severity of PMS symptoms and improve individuals' quality of life. Therefore, it is suggested that HRW consumption could be considered as an option for reducing the severity of PMS symptoms. HRW consumption is a simple practice that individuals can easily perform in their homes or workplaces. This method is believed to be easily applicable by everyone, practical, promoting individual responsibility for health, and cost-effective. Hence, it is recommended that nurses and other healthcare professionals consider the use of HRW in helping individuals cope with PMS and improving their quality of life.

Limitations: Participants could not be followed for over two menstrual cycles after completing the intervention. Therefore, the long-term effectiveness of the intervention could not be evaluated. The study participants were recruited from a single center and consisted of nursing students. Thus, the generalizability of the findings to other populations may be a limited issue. Therefore, it is recommended that more extended follow-up studies be conducted in broader populations.

## Data Availability

The data supporting this study's findings are available on request from the corresponding author.

## Abbreviations

PMS: Premenstrual syndrome
GABA: Gamma-amino butyric Acid
H_2_: Molecular hydrogen
HRW: Hydrogen-rich water
